# Examining rural health equity and impact through the translational science benefits model: outcomes from the CTSA Consortium of Rural States (CORES)

**DOI:** 10.3389/fpubh.2025.1538494

**Published:** 2025-04-28

**Authors:** Maggie Padek, Rebecca Butcher, Rebecca deLacerda Allen, Hilary L. Surratt, Maran Subramain, Beth Tigges, Alyson G. Eggleston, Jessica H. Presley, Trent Matheson, Nasser Sharareh

**Affiliations:** ^1^Frontiers Clinical and Translational Science Institute, University of Kansas Medical Center, Kansas City, KS, United States; ^2^Dartmouth SYNERGY Clinical and Translational Science Institute, Geisel School of Medicine, Lebanon, NH, United States; ^3^Center for Clinical and Translational Science, University of Kentucky College of Medicine, Lexington, KY, United States; ^4^Institute for Clinical and Translational Science, University of Iowa, Iowa City, IA, United States; ^5^University of New Mexico Health Sciences Clinical and Translational Science Center, Albuquerque, NM, United States; ^6^Clinical and Translational Science Institute, Pennsylvania State University, Hershey, PA, United States; ^7^The Translational Research Institute, University of Arkansas for Medical Sciences, Little Rock, AR, United States; ^8^Utah Clinical and Translational Science Institute at the University of Utah, Salt Lake City, UT, United States; ^9^Department of Population Health Sciences, Spencer Fox Eccles School of Medicine, University of Utah, Salt Lake City, UT, United States

**Keywords:** rural health, translational science, TSBM, impact evaluation, CTSA

## Abstract

**Introduction:**

Rural communities often lack access to healthcare, have limited resources and infrastructure, and may experience suboptimal translation of evidence-based interventions into practice or measurement of translational research impact. The Consortium of Rural States (CORES), comprising eight Clinical and Translational Science Award (CTSA) hubs, is a research consortium that focuses on clinical and translational research impacting rural health.

**Methods:**

Utilizing the Translational Science Benefits Model (TSBM) framework, each CTSA hub’s evaluation lead co-created an inventory of rural-focused activities, projects, and initiatives that occurred at their respective site during the funding period 2021–2023. Variables included program area; activity type and description; target population; activity status; outputs; and short-term outcomes. The evaluators then mapped site outcomes according to the four TSBM domains (clinical, community, economic, policy) and 30 subcategories (benefits).

**Results:**

184 rural-focused activities, projects and initiatives were identified across the hubs. All rural-focused efforts involved impacts in the Community and Clinical domains of the TSBM, with >60% focusing on Community impacts. These results suggest an opportunity gap to better define Economic and Policy-level impacts in the context of rural-focused initiatives.

**Discussion:**

This work demonstrates a novel mapping of the TSBM to rural health research settings and explores the nuances of using the concepts and domains of the TSBM as a coding tool. This work gives the Consortium insight on the types of projects and impacts that are supported and how to prioritize more exploration of the full range of translational science benefits in rural health initiatives going forward.

## Introduction

Disparities in health outcomes between urban and rural settings are well documented within the scientific literature (1–3). Overall, rural residents, who account for 14% of the U.S. population, experience significantly higher rates of mortality and chronic diseases than urban residents (4, 5). Differential health outcomes in rural communities are largely attributed to a variety of social determinants of health, such as poverty, limited access to healthcare, inadequate resources and infrastructure for health services (6, 7), and reduced educational and economic opportunities (6, 7). Rural communities may also experience suboptimal translation of evidence-based health interventions into practice.

Moreover, there are several challenges in both conducting rural health research and describing associated impacts. Challenges range from agreement on how rurality is defined to dealing with methodological issues unique to recruiting and studying health issues in socially and culturally diverse and geographically dispersed populations (8, 9). Over the last decade, a number of frameworks to measure research impacts have been developed (10) with attempts to create specific frameworks to measure rural health research impacts (11) but it is unclear the degree to which these frameworks are widely accepted and utilized. Disseminating rural health research findings have additional challenges ensuring that results are accessible to non-researchers and presented in a way that resonates with communities that have grown increasingly culturally, socially and economically diverse (8, 12).

These challenges led the National Center for Advancing Translational Science (NCATS) at the National Institutes of Health (NIH) to present a report in 2019 emphasizing the need for translational science to address rural health inequities (13). Since then, there have been research and dissemination initiatives focused on improving equitable care access in rural communities but limited attention on the impact of this focus on rural health (13). In 2012, the Consortium of Rural States (CORES)^1^ formed as an informal working group of academic medical institutions, funded by smaller Clinical and Translational Science Awards (CTSAs) from NCATS, with a shared public health focus on rural populations (14, 15). CORES institutions collaborated to reduce the burden of illness and mortality within rural populations through inter-institutional pilot funding and sharing best practices. The membership of CORES includes hubs with current or recent CTSA grants in the G and T funding tiers (small to small-medium CTSAs). The limitation of CORES members to small to small/medium CTSA hubs is intended to bring together hubs with similar resources and with a similar impact on their respective institutions. By 2024, the Consortium had grown to eight institutions: Dartmouth SYNERGY Clinical Translational Science Institute (CTSI), Pennsylvania State University CTSI, Translational Research Institute at the University of Arkansas for Medical Sciences, University of Iowa Institute for Clinical Translational Science (ICTS), Frontiers CTSI at University of Kansas, University of Kentucky Center for Clinical and Translational Science (CCTS), University of New Mexico Health Sciences Clinical and Translational Science Center (CTSC), and University of Utah CTSI. The Consortium has funded over 34 pilot projects totaling more than $2.1 million since its inception. CORES convenes annually at a rotating host institution to share advancements and best practices from their respective institutes and to collaborate on initiatives within smaller workgroups. Despite the longevity of CORES, prior to 2023, there had been no collective effort to study shared and divergent rural public health initiatives and challenges, nor to quantify the impact domains of rural research activities within these hubs.

The Translational Science Benefits Model (TSBM) was developed in 2016 by Luke et al. at Washington University in St. Louis (16, 17) to characterize the impacts of translational science activities from CTSA-funded projects across four domains: Clinical and medical (clinical), Community and public health (community), Economic, and Policy and legislative (policy). Within those four domains, the authors identified 30 health and social benefits that serve as intermediary benchmarks to assess health and social impact. Together, these domains and intermediary benchmarks provide a framework for understanding the long timespan between initiating translational science activities and measuring realized health and social benefits. The TSBM has been widely adopted by evaluators across CTSAs and there are multiple calls in both the literature (18, 19) and at the federal funding level (20, 21) to utilize the TSBM in varied ways to describe the impacts of translational science research. Although the TSBM has been utilized in logic model planning (22), capacity assessments (23), project assessment (24, 25), and case study development (17), few efforts have utilized the framework to study health impacts in rural contexts (26, 27). The TSBM is poised to describe research impacts in rural settings as its framework is anchored in descriptive definitions that are meant to be understood by the lay public (8, 17). The aim of this collaborative study was to apply the TSBM framework in a novel way to inventory and classify the activities and outcomes of the rural-focused research and scholarship initiatives supported by the CORES hubs to (1) explore the range of benefits across the Consortium, (2) identify any gaps in utilizing the TSBM framework, and (3) contribute to advancing methods grounded in translational science benefits for use across the wider CTSA community.

## Methods

The CORES evaluation workgroup consisted of at least one evaluation representative from each hub. The study was confined to the eight CTSA hubs represented by each CORES institution in the workgroup, which ensured availability of data aligned with the rural focus of the project. The group met monthly from September 2023 to September 2024 to develop project aims and to achieve consensus on study design, review methods, inclusion criteria, and data points to be gathered. Smaller working groups formed throughout the project to accomplish specific tasks such as finalize the data collection inventory tool, conduct data analysis, and synthesize results. Hubs submitted the requisite information to their respective Institutional Review Boards, and all were designated as not human subjects’ research and exempt from IRB approval.

### Sampling and data collection

Hubs independently compiled relevant data for the 2021–2023 inventory over a four-month period utilizing prior years’ Research Performance Progress Reports (RPPRs), data from pilot program awards with rural foci, as well as surveys of local staff, scholars, and faculty and/or querying relevant investigators at local team meetings. Since each hub engages in unique activities and captures output data differently, the representative evaluator selected source documents best positioned to contain the data needed to inform the inventory. Activities and projects were included in the inventory if they met the following criteria: (a) demonstrated focus on rural health settings and populations; (b) were funded through the CTSA; and (c) project status was ‘in progress’ or ‘completed’ during the analysis time-period. Projects and activities were excluded if they had not yet started or were limited to brief, transactional activities (e.g., one-time assistance on a project such as statistical consultations).

**Figure 1:**
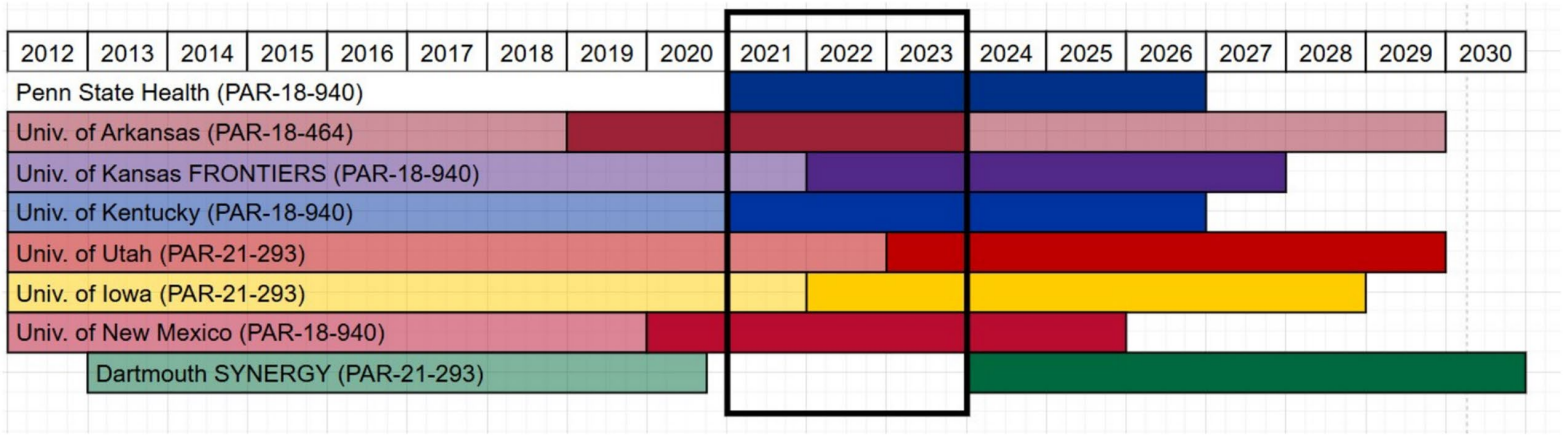
Period of analysis (2021–2023) relative to CORES’ current CTSA funding.

Because each hub was on a different timeline for their current grant cycle (Figure 1), the team restricted the analysis to activities and projects occurring during 2021–2023, as seven of eight CORES sites were actively funding pilot projects and programs during this overlapping time window. All hubs contributed data apart from Dartmouth as they were unfunded during the time period defined. Their evaluator was still an active contributor in the study design, data cleaning and data analysis. Data were compiled in the inventory, a shared Microsoft Online Excel spreadsheet hosted on a restricted access Microsoft Teams channel with each hub having a standardized worksheet to complete. Fields were defined with a codebook, and dropdown menus and other data validation were used to standardize data entry. Monthly meetings with all evaluators were held to ensure data entry consistency and to resolve and troubleshoot any discrepancies in how data was entered and interpreted.

### Variables

Using the TSBM conceptual framework as a guide, the team identified the key variables that were (a) descriptive of the projects/activities, (b) relevant for impact measurement, (c) tracked by hubs, and (d) feasible to compile and report in the collection period. Variables included the activity title and description, the population of focus, status of the project, and judgments on the perceived benefit of each project according to TSBM definitions. The table included in the Supplementary materials provides a thorough outline and description of each variable along with how evaluators categorized and scored them. Benefits were also defined as either “potential” meaning there was no demonstrated evidence of achieving that benefit, but the evaluator believed the research could generate the outcome in the future and “demonstrated” meaning there was documented evidence that the benefit was already achieved by the research (usually through completion report of study or publication).

### Data analysis

Once all data were entered into the shared spreadsheet, a subcommittee of three members (MP, RB, RDA) cleaned and checked the data, identified any missing data, and developed a data analysis strategy. Each hub’s data was double coded by two different coders as a form of investigator triangulation. Coders did not code their own hub’s data. Coders met over three separate meetings to align coding approach, discuss any discrepancies in the data, and reach consensus on final scoring. Some hubs were not tracking TSBM variables during the defined time-period and did not have data available for variables such as “potential” or “demonstrated” and “level of impact.” Missing data and questions were directed back to the relevant hub evaluator to resolve, if possible. Not all missing data could be identified, and missing data were excluded from analysis. A new variable was created to code primary and secondary target populations from the free text entry to standardize population focus. Counts and averages were calculated for TSBM benefits per project/activity. Variable counts were calculated across all hubs together and for each hub independently, and averages were computed when relevant. Program areas associated with the projects were identified by hub and thematically analyzed across all hubs. Differences in program area terminology were observed across hubs, mostly attributable to the language used in the Notice of Funding Opportunity (NOFO) under which each hub was operating (PARs are shown in Figure 1). The team used inductive analysis to create thematically common program area categories for summarization. Final coded data were recorded in a separate worksheet designated for each coder, and one coder (RDA) compiled all data into a final analysis worksheet.

## Results

Across the seven included hubs, a total of 184 projects and activities focusing on rural health and populations met the inclusion criteria. On average, there were 26 projects per hub (range: 19–48). Projects spanned 10 different programmatic areas including pilot funding programs, K & T scholar programs, quality improvement activities, research capacity and methods support, workforce development, and community engagement activities. Table 1 presents the hub-level and aggregate counts of rural-focused research activities, translational science (TS) benefits and level of impact, and associated target populations collected and scored with the TSBM inventory.

**Table 1:** Hub-level and aggregate counts of CORES rural research activities (TSBM inventory results).

Variable	Total count across all sites	Average across all sites	Hub 1 totals	Hub 2 totals	Hub 3 totals	Hub 4 totals	Hub 5 totals	Hub 6 totals	Hub 7 totals
# of CTSI program areas with rural research activities	N/A	6.6	3	8	10	4	5	10	6
# of rural research activities or projects	184	26.3	48	23	24	19	19	31	20
# of completed projects to date	62	8.9	18	1	4	3	10	17	9
# of projects in progress	119	17.0	30	22	19	15	8	14	11
Avg # of TS benefits per project/site*	14.14	2.4	1.29	1.23	1.57	3.78	1.9	^	4.37
Total # of Clinical Domain projects	94	13.4	22	16	10	13	7	14	12
Total # of Community Domain projects	120	17.1	28	10	23	12	18	16	13
Total # of Economic Domain projects	9	1.3	2	0	1	1	2	0	3
Total # of Policy Domain projects	8	1.1	1	1	3	2	0	1	0
# of POTENTIAL benefits (across all domains)	107	21.4	^	23	25	21	29	^	9
# of DEMONSTRATED benefits (across all domains)	37	7.4	^	2	10	5	7	^	13
Population focus of the CORES projects									
Patient/Individuals	89	12.7	40	7	2	9	14	7	10
Providers/Clinical Staff/ CHWs	46	6.6	8	5	9	2	9	5	8
Researchers/Res Admin & Staff	49	7.0	5	6	11	6	2	17	2
Community Org	75	10.7	7	12	12	7	1	23	13
Level of impact of CORES rural research activities									
# of individual-level impacts	8	1.6	^	0	1	7	0	^	0
# of local-level impacts	10	2.0	^	2	1	1	6	^	0
# of organization-level impacts	9	1.8	^	8	1	0	0	^	0
# of state-level impacts	58	11.6	^	4	6	15	12	^	21
# of regional-level impacts	42	8.4	^	5	24	8	3	^	2
# of national-level impacts	34	6.8	^	8	5	2	16	^	3

^ Data missing from Hub.

* Each project could align with more than one translational science benefit (there are 30 benefits in the TSBM).

**Figure 2:**
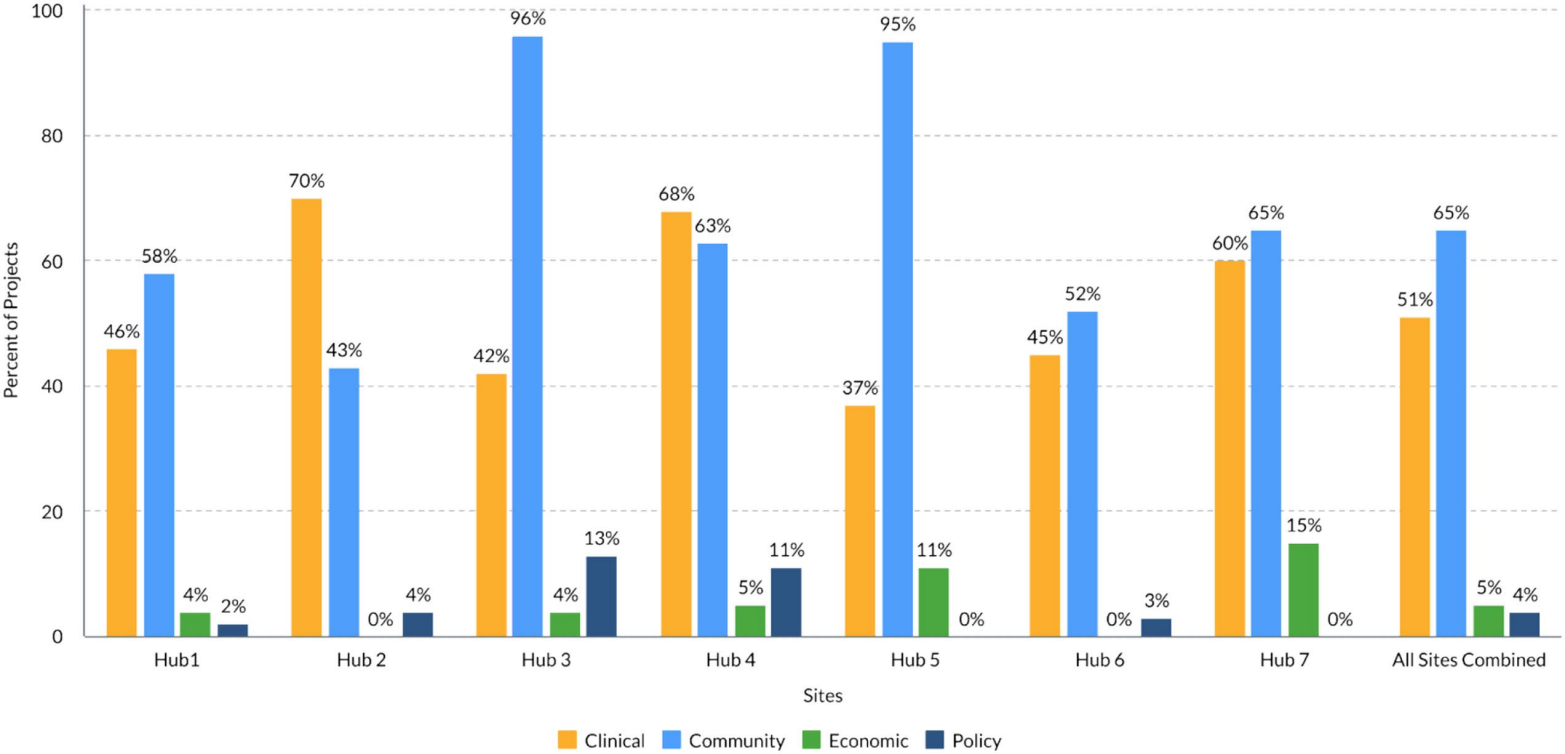
CORES site projects by TSBM domains.

Figure 2 summarizes the distribution of TSBM benefits by domain associated with the research activities of each of the seven hubs in the analysis. On average, there were 2.4 (range 1.2–4.4) TSBM benefits per project per hub and three times as many *potential* TSBM benefits reported as *demonstrated* TSBM benefits (five hubs reporting). Across all hubs, 65% of the projects mapped to Community benefits, 51% mapped to Clinical benefits, 5% mapped to Economic benefits and 4% mapped to Policy benefits. The largest proportion (48%) of projects focused on patients or individuals as the population of benefit, followed by projects which focused on community organizations as the population of benefit (41%). Most projects or rural research activities across the seven hubs achieved state level impact (59%), followed by regional impact (42%) and impact at a national level (34%). Importantly, most projects’ impacts (66%) were still considered “potential,” as the TSBM benefits were not yet demonstrated at the time of data collection.

## Discussion

Overall, the results showed that the TSBM offered a useful framework to code and analyze a wide variety of rural-focused projects and enabled a cross-site exploration of demonstrated and potential outcomes, which to our knowledge has not been done before. It was expected that rural focused projects would largely map to the Clinical or Community TSBM domains. This finding is consistent with other studies that have mapped the TSBM to outcomes (28); rural-focused initiatives are typically designed to impact under-resourced communities with clinical and community-based interventions. For instance, a Clinical domain study that took place in rural New Mexico focused on prescriber uptake of the innovative biomedical technology of the ECHO program to treat opioid use disorders (29). An example of a Community domain study was the expansion of genomic testing services into rural Kansas primary care clinics to improve healthcare accessibility and healthcare delivery (30). These projects highlight how rural CTSAs impact rural healthcare by improving access and capacity in rural areas, and how these activities are aligned with the TSBM framework.

However, very few projects mapped onto the Economic and Policy domains. This may be because the benefits within Economic and Policy domains are typically actualized several years after a project has concluded and may not be the typical target outcomes for projects focused on rural health (31). Further compounding this issue, very few hubs have established evaluation measures to track economic or policy impacts (32) and investigators may not even consider what future economic and policy outcomes they expect to see in initial project designs. Many CTSAs and investigators do not have the capacity or expertise to measure economic benefits such as cost savings, cost effectiveness, and the societal and financial cost of illness, three of the Economic subdomains. Economic analyses could be encouraged and included in CTSA-funded projects, and CTSAs could offer economic analysis as core services for investigators.

The skewedness of the observed benefits toward Clinical and Community TSBM domains may also stem from how the economic and policy benefits are defined, as the definitions may lack sufficient detail. The noted benefits within these two domains of the TSBM are broad and more distal as research outcomes (e.g., policies, legislation, patents, license agreements, cost savings). Future work is needed to better define and potentially expand the TSBM benefits within the Economic and Policy domains to characterize a wider range of economic and policy outcomes relevant to underserved populations like rural communities—areas that have been historically marginalized and excluded from economic gains and lacking advocates among policymakers. Similarly, further clarification of policy benefits could help researchers understand appropriate targets for systems and population health research (33). This has been noted as an area of need specific to rural health services research (34).

Unfortunately, policy changes take time and can be difficult to track—as the market demand for a product like Overton (35) proves. Overton is a cloud-based application that aims to identify influences of research on policy through real-time tracking of state and federal policy actions linked to scientific research citations which may be useful for tracking how research activities impact policy. However, legislative, and even many policy statements rarely include exhaustive lists of scientific references as the basis for their scientific decisions, often making it difficult to track these linkages even if they do exist. Local policy impacts may be difficult to detect if not reported by investigators or scientific results are undervalued by policy makers.

Although there were fewer Economic and Policy benefits than Community and Clinical, all four domains are critical for fulfilling the promise of translational science. To increase the Economic and Policy benefits, better upstream consideration and integration of these benefits and the pathways to achieve them are needed. Better operationalization of the specific activities involved in such benefits would be an important first step to understanding the potential Economic and Policy benefits of translational science along with discussion of how these may differ between rural and urban settings and to what extent definitions capture beneficial activities in varying communities. Currently, beneficial activities and outcomes include patents, technology transfer, and workflow/procedural enhancements. Other activities could include cost–benefit analyses, legislative or advocacy efforts, and social media efforts to extend health impacts. Consideration of all activities and benefits and their applications to rural health research will also be important as the field progresses.

However, there were challenges applying the framework as a coding tool when the benefits within each TSBM domain lacked sufficient specificity. While Luke et al. have provided the definitions of each TSBM benefit, there is little guidance on what documentation is necessary for an impact to align with a benefit and what constitutes a benefit as “demonstrated” (36). Most of the CORES’ institutions had not previously applied the TSBM to their own assessment of rural projects prior to this study, leading evaluators to retrospectively code TSBM domains and benefits for each research project. This resulted in instances of subjective coding decisions which required multiple rounds of review and discussion among the analysis subcommittee to reach standardization of coded benefits across projects and sites. However, this work demonstrates that the TSBM can be utilized as a coding tool rather than strictly for case study development. It has also been suggested that the TSBM should be expanded to integrate more health equity concepts (37, 38). If broadening the scope of the TSBM to include health equity concepts, it will be important to consider rural populations. The current inclusion of healthcare access in the Community domain likely does not fully encompass a wider range of health equity impacts on rural populations, such as health literacy, food availability, transit options, and place-based environmental impacts on health.

### Limitations

Although this project was intentionally designed as a cross-hub analysis using data from multiple CTSA hubs, there were some limitations to our approach. First, hubs varied in administrative, funding and evaluation structures which resulted in a relatively narrow timeframe and inclusion criteria for the data. The analysis period was limited to 3 years, although some hubs had been collecting data for much longer than others. Sampling decisions were compounded by whether to include the full range of CTSA-funded activities or only those where potential translational science benefits could be tracked and recorded. We decided not to include brief t*ransactional* activities, such as informatics or methodology consultations to ensure more consistency in data across the hubs, although some hubs provided such services to rural-based providers or in settings that clearly benefited from new data knowledge and access. Documentation of demonstrated benefits was subjective to each hub and was not included in this analysis. Future applications of the TSBM inventory will benefit from potentially broader inclusion criteria.

For this study, each site collected data in an Excel spreadsheet. In the future, a more structured format of data collection (e.g., REDCap project) would help standardize the process, validate fields, and reduce missing data and non-valid free text entries. Additional changes to the data fields may also be warranted, including allowing the entry of multiple benefits per domain and allowing multiple populations within the same subgroup (e.g., different providers, different patient populations). As noted previously, there is also a need to increase understanding of when a benefit has been ‘demonstrated’ rather than still considered a ‘potential’ benefit. Future alignment of evaluation and metric tracking will provide more robust data than the current retrospective analysis. A final limitation is the potential impact of the COVID-19 pandemic on the study timeframe (2021–2023). Because the pandemic negatively impacted many research projects by disrupting activities, data collection, and timelines, it is possible that research occurring proximate to the pandemic had limited reach and outcomes resulting in fewer observed TSBM benefits.

### Implications for rural health projects

Despite some of these limitations, the application of the TSBM in this study was valuable in providing a shared framework for identifying and describing health research impacts among distinct and regionally diverse institutions. It was particularly useful as an initial approach to examining rural health impacts, as this study was the first recorded attempt to explore translational science impacts in rural communities across multiple states. The TSBM provides a basis for identifying what is currently known and highlighting gaps that need additional study. With additional research, there can be a holistic evaluation of the impact of rural-focused research on the greater public health that is informed by the TSBM and other complementary approaches (39).

### Next steps

Further refinements of the TSBM as described above will enable easier and more robust applications of the model to assess impacts of future projects within the Consortium. Indeed, better defined inclusion criteria coupled with ‘real-time’ tracking of the TSBM benefits and, enhanced data collection methods will strengthen cross-hub data analyses in the future. In addition, using examples of economic and policy benefits emerging from this and future analysis, new measures and evaluation processes can be developed to better track these impacts, even while recognizing the variability in actualization time. It will be important moving forward to have collaborations in standardizing measures and compiling data with non-rural focused hubs, which would afford a better understanding of differences between rural-serving and urban-serving hubs. Finally, the use of cutting-edge tools, such as Overton or other artificial intelligence or machine learning tools, could supplement current practices in identifying and defining not only policy benefits within and across projects but facilitate more efficient identification of demonstrated outcomes from these projects in the literature (40). An additional future step is further exploring these demonstrated outcomes through qualitative analysis to align our work within the original application of the TSBM through case study development.

## Conclusion

The TSBM is a compelling framework for describing impacts of translational science research. However, the application of the framework in impact assessments is still in development and being tested by many groups and in many different research settings. The CORES Consortium wanted to test the applicability of the TSBM to rural health research settings and explore the nuances of using the domains and benefits of the TSBM as a coding tool rather than a case study tool. This work highlights where refinement of the tool is still needed in applying the model to assess within-and across-hub impacts. But this work also gives the Consortium insight on the types of projects and impacts that are currently supported and how to prioritize more exploration of the full range of translational science benefits in rural health initiatives going forward. The CTSAs can be influential drivers of research focus and priorities at their respective institutions. While CTSAs are specific research infrastructure to the United States, we believe this application of the TSBM framework to rural research projects can be applied to other assessments of research impacts in rural settings both within the United States and across international settings. By demonstrating the impact of hubs’ work on rural health, together the CORES hubs can push toward alleviating health disparities within these communities.

## Data Availability

The raw data supporting the conclusions of this article will be made available by the authors, without undue reservation.

## Footnotes

^1^ https://www.ctsacores.com/
