# Supplementary material for: Examining rural health equity and impact through the translational science benefits model: outcomes from the CTSA Consortium of Rural States (CORES)
Source: Front Public Health. 2025 Apr 28;13:1538494. doi: 10.3389/fpubh.2025.1538494 (PMC12066754; doi:10.3389/fpubh.2025.1538494)
Supplement: Supplementary file 1 [file Table_1.docx]

**Supplemental Materials**

**Table 1. Variables**

| **Variable** | **Description** | **Value** |
| --- | --- | --- |
| Core/Element | Indicate the programmatic area, Element or module leading/sponsoring this activity/project | Free Text Entry |
| Activity | Provide the title of the activity. If this is a pilot project, list the title of the project in quotes. Activities should be discrete projects that were directly supported by the CTSI. Transactional activities (such as one-time consultations) were to be excluded. | Free Text Entry |
| Activity description | Provide 1-2 sentence description of the activity. Describes how it's new or improved. | Free Text Entry |
| Primary Population focused | Describe the primary population focus | Free Text |
| Secondary Focus Population | If applicable, describe the secondary population focus | Free text |
| Status | Current status of the activity | Forced Choice: *Not Started, In Progress, Completed* |
| Metrics/ Outputs | Key performance indicators that are being measured for this activity. | Free Text |
| Short-Term  Outcomes | Describe any short-term outcomes from this activity. | Free Text |
| TSBM Domains_1 | Map the outcomes against one of the four domains from the Translational Science Benefits Model | Forced Choice *Clinical, Community, Economic, Policy* |
| TSBM Benefit(s)_1 | List the specific benefit(s) for the specified domain as it aligns with the outcomes | Referenced TSBM website for corresponding list <https://translationalsciencebenefits.wustl.edu/benefits/> |
| Description of TSBM Benefit_1 | 1-2 sentences describing how the outcome aligns with the stated TSBM Benefits | Free Text |
| Level of Impact_1 | Indicate which level of impact the benefit has been or will be demonstrated. | Forced Choice: *Individual, Local, Organizational, State, Regional, National* |
| TSBM Status_1 | Indicate whether this benefit has been demonstrated and documented or if the benefit is anticipatory | Forced Choice: *Demonstrated*  *Potential*  *Nearly Demonstrated* |
| TSBM Domains_2 | Map the outcomes against one of the four domains from the Translational Science Benefits Model | Forced Choice: *Clinical, Community, Economic, Policy* |
| TSBM Benefit(s)_2 | List the specific benefit(s) for the specified domain as it aligns with the outcomes | Referenced TSBM website for corresponding list <https://translationalsciencebenefits.wustl.edu/benefits/> |
| Description of TSBM Benefit_2 | 1-2 sentences describing how the outcome aligns with the stated TSBM Benefits | Free Text |
| Level of Impact_2 | Indicate which level of impact the benefit has been or will be demonstrated. | Forced Choice: *Individual, Local, Organizational, State, Regional, National* |
| TSBM Status_2 | Indicate whether this benefit has been demonstrated and documented or if the benefit is anticipatory | Forced Choice: *Demonstrated*  *Potential*  *Nearly Demonstrated* |
| TSBM Domains_3 | Map the outcomes against one of the four domains from the Translational Science Benefits Model | Forced Choice: *Clinical, Community, Economic, Policy* |
| TSBM Benefit(s) | List the specific benefit(s) for the specified domain as it aligns with the outcomes | Referenced TSBM website for corresponding list <https://translationalsciencebenefits.wustl.edu/benefits/> |
| Description of TSBM Benefit_3 | 1-2 sentences describing how the outcome aligns with the stated TSBM Benefits | Free Text |
| Level of Impact_3 | Indicate which level of impact the benefit has been or will be demonstrated. | Forced Choice: *Individual, Local, Organizational, State, Regional, National* |
| TSBM Status_3 | Indicate whether this benefit has been demonstrated and documented or if the benefit is anticipatory | Forced Choice*: Demonstrated*  *Potential*  *Nearly Demonstrated* |
| TSBM Domains_4 | Map the outcomes against one of the four domains from the Translational Science Benefits Model | Forced Choice: *Clinical, Community, Economic, Policy* |
| TSBM Benefit(s)_4 | List the specific benefit(s) for the specified domain as it aligns with the outcomes | Referenced TSBM website for corresponding list <https://translationalsciencebenefits.wustl.edu/benefits/> |
| Description of TSBM Benefit_4 | 1-2 sentences describing how the outcome aligns with the stated TSBM Benefits | Free Text |
| Level of Impact_4 | Indicate which level of impact the benefit has been or will be demonstrated. | Forced Choice: *Individual, Local, Organizational, State, Regional, National* |
| TSBM Status_4 | Indicate whether this benefit has been demonstrated and documented or if the benefit is anticipatory | Forced Choice: *Demonstrated*  *Potential*  *Nearly Demonstrated* |

**Table 2. Data Analyzed**

| **Data Analyzed** | **Original Variable** | **Process/Notes** |
| --- | --- | --- |
| # of program area domains | Core/Element | Counted each original program area listed |
| List of programmatic areas | Core/Element | Listed out specific programming areas to recode into defined categories |
| # of activities/projects | Activity | Total count of all activities listed on spreadsheet |
| # of completed projects/activities to date | Status | Count of total projects that have “completed” status |
| # of projects/activities in progress | Status | Count of total projects that have “in progress” status |
| Count of TSBM benefits | TSBM Benefits_1, TSBM Benefits_2, TSBM Benefits_3, TSBM Benefits_4 | Total number of TSBM benefits counted across all projects at each hub |
| Average # of TSBM benefits per project | TSBM Benefits_1, TSBM Benefits_2, TSBM Benefits_3, TSBM Benefits_4 | Average number of TSBM benefits listed for each project, divided by the total number of activities listed |
| Total # of Clinical Domains projects | TSBM Domains_1, TSBM Domains_2, TSBM Domains_3, TSBM Domains_4 | Count across all projects the amount that ‘Clinical” appears across those four variables |
| Total # of Community Domains projects | TSBM Domains_1, TSBM Domains_2, TSBM Domains_3, TSBM Domains_4 | Count across all projects the amount that ‘Community’ appears across those four variables |
| Total # of Economic Domains Projects | TSBM Domains_1, TSBM Domains_2, TSBM Domains_3, TSBM Domains_4 | Count across all projects the amount that ‘Economic’ appears across those four variables |
| Total # of Policy Domains Projects | TSBM Domains_1, TSBM Domains_2, TSBM Domains_3, TSBM Domains_4 | Count across all projects the amount that ‘Policy’ appears across those four variables |
| Total # of ‘potential’ TSBM projects | TSBM Status_1, TSBM Status_2, TSBM Status_3, TSBM Status_4 | Total count across all activities and TSBM domains that are listed as “potential.” This count could be higher than the total # of activities since each activity could have more than one TSBM domain |
| Total # of ‘demonstrated’ TSBM projects | TSBM Status_1, TSBM Status_2, TSBM Status_3, TSBM Status_4 | activities and TSBM domains that are listed as “demonstrated.” This count could be higher than the total # of activities since each activity could have more than one TSBM domain |
| Total # of ‘patient/individual’ population focus | Primary Population Focus & Secondary Population Focus | Primary and secondary population focus variables were recoded from free text to the categories: “individual/patient, provider/clinical staff, research staff, community organizations” |
| Total # of ‘Providers/Clinical Staff/CHWs’ population focus | Primary Population Focus & Secondary Population Focus | Primary and secondary population focus variables were recoded from free text to the categories: “individual/patient, provider/clinical staff, research staff, community organizations” |
| Total # of ‘Researchers / Res Admin & Staff’ population focus | Primary Population Focus & Secondary Population Focus | Primary and secondary population focus variables were recoded from free text to the categories: “individual/patient, provider/clinical staff, research staff, community organizations” |
| Total # of ‘Community Orgs’ population focus | Primary Population Focus & Secondary Population Focus | Primary and secondary population focus variables were recoded from free text to the categories: “individual/patient, provider/clinical staff, research staff, community organizations” |
| # of individual-level impacts | Level of Impact_1, Level of Impact_2, Level of Impact_3, Level of Impact_4 | Count across all activities the # of individual-level TSBM impacts |
| # of local-level impacts | Level of Impact_1, Level of Impact_2, Level of Impact_3, Level of Impact_4 | Count across all activities the # of local-level TSBM impacts |
| # of organization-level impacts | Level of Impact_1, Level of Impact_2, Level of Impact_3, Level of Impact_4 | Count across all activities the # of organization-level TSBM impacts |
| # of state- level impacts | Level of Impact_1, Level of Impact_2, Level of Impact_3, Level of Impact_4 | Count across all activities the # of state-level TSBM impacts |
| # of regional-level impacts | Level of Impact_1, Level of Impact_2, Level of Impact_3, Level of Impact_4 | Count across all activities the # of regional-level TSBM impacts |
| # of national-level impacts | Level of Impact_1, Level of Impact_2, Level of Impact_3, Level of Impact_4 | Count across all activities the # of national-level TSBM impacts |
